# *Homo sapiens*, Chimpanzees and the Enigma of Language

**DOI:** 10.3389/fnins.2019.00558

**Published:** 2019-05-31

**Authors:** Ambrosio Bermejo-Fenoll, Alfonso Panchón-Ruíz, Francisco Sánchez del Campo

**Affiliations:** ^1^Department of Oral Medicine, University of Murcia, Murcia, Spain; ^2^Department of Physical Medicine, University of Alicante, Alicante, Spain; ^3^Department of Histology and Anatomy, Miguel Hernández University, Elche, Spain

**Keywords:** *sapiens*, chimpanzees, language, oral cavity, mandible, evolution, craniometric parameters

## Abstract

**Objectives:**

The present study explores the hypothesis that the anatomical bone structures of the oral cavity have probably evolved under the influence of language function. The possible changes have been evaluated by comparing two close species essentially differentiated from each other by spoken language.

**Materials and Methods:**

Twenty dry skulls and 20 mandibles of modern Caucasians were compared with 12 dry skulls and 12 mandibles of chimpanzees, with the analysis of 37 variables and the definition of new anatomical parameters.

**Results:**

A number of highly significant differences were found between humans and chimpanzees. The human temporomandibular joint is comparatively less flat and has a more limited excursive movement range, with structural elements that seem to be lighter. A significant difference is noted in mandibular alveolar vergency and in the internal slope of the mandibular symphysis where the oral cavity’s morphology is modified, thereby increasing the free space for tongue movements in humans. The chin, which is unique to the human species, is quantified through the external slope of the mandibular symphysis with a lesser angle in humans.

**Discussion:**

It is obvious that there are differences between humans and chimpanzees in the bone morphology of the oral cavity structures. This has been confirmed with the analysis of new variables. Together with other factors (bipedalism, habits, and genetics) speech in humans must have played an important role in the aforementioned differences between humans and chimpanzees. The number of mandibular movements involved in speech is far greater than those used in chewing, which must have conditioned the evolution of the oral structures implicated in the development of language. On average, humans weigh 70 kg and chimpanzees 44 kg. However, the majority of the variables studied in skulls and mandibles are greater in chimpanzees, which suggests that the evolution of the oral zone in humans has suffered a reduction in size with changes in shape. The refinement of the supralaryngeal vocal tract in the human species must have co-evolved with speech fairly recently. The human skull has temporomandibular joints that are comparatively less flat with a more limited movement. There is a greater lingual space and there is also a chin that suggests a muscular stimulant. This leads to the conclusion that, at least in part, speech is behind all these changes, although it is difficult to establish a cause-effect relationship.

## Introduction

“Of all animals, only Man possesses speech” (Aristotle). In effect, speech is a characteristic exclusive to *Homo sapiens*, though the human phonatory apparatus is not a specific organ. Mammals have had a larynx, pharynx, and oral cavity for many millions of years and these structures allow the different species to communicate.

The present study involves a comparative anatomical investigation of chimpanzee facial bones vs. human facial bones.

Although the difference in body mass between humans and chimpanzees is always in favor of humans, the maxillofacial massif appears larger and more prominent in chimpanzees.

The working hypothesis of this study is that the bone structures that participate in phonatory function have evolved conditioned among other factors to the specific movements of spoken language, and with the purpose of facilitating such movements.

By studying the bone structures of the temporomandibular joints (mandibular condyle and articular tubercle of the temporal bone), the bone structures surrounding the tongue (alveolar process, mandible, and hard palate) and the hard structures related to the lower lip (chin and its inclinations), it may be possible to establish associations with speech.

In addition to language three conditioning factors could have influenced the development and evolution of the hard structures relating to the oral cavity of modern man: bipedalism, oral habits, and genetics.

### Bipedalism

Bipedalism resulted in a series of adaptive phenomena that would later contribute to the appearance of the spoken language. The relation between the larynx, pharynx, and oral cavity changed. The upright position allowed mandibular horizontalization, lengthening of the pharynx and descent of the larynx. The chimpanzee’s epiglottis covers the ventral side of the oropharynx, overlapping and extending over and behind the free edge of the soft palate. This is similar to what occurs in a lactating human. After the chimpanzee’s birth, the larynx descends as occurs in children, but the hyoid bone does not (Nishimura et al., 2006). The pharynx in adult humans is much longer than in children and the hyoid bone and larynx descend (Lieberman et al., 2001), eliminating the closure between the epiglottis and the soft palate.

Compared to a lactating human or a chimpanzee, the descended pharynx of an adult has the acoustic significance of producing sounds (Lieberman et al., 2001) as the vocal tract includes the pharyngeal, oral and nasal cavities.

### Oral Habits

Anomalous oral habits prolonged over time have altered craniofacial development and growth. They form part of the environmental factors capable of modifying these structures (Shanhraki et al., 2012).

The tongue must be perfectly positioned in relation to the palate for the palate and dental arches to develop correctly. When this does not occur, particularly during infantile development, the palate curves (ogival palate) and the occlusion is altered (anterior open bite and posterior crossbite). The middle and lower thirds of the face change and asymmetries frequently appear. These are the consequences. The causes: breathing through the mouth and atypical separation of the upper and lower teeth with a finger, tongue, or lower lip.

As indicated in the Section “Materials and Methods”, specimens outside the parameters of normality with malocclusion, deformities and atypical or asymmetric palates were rejected.

In connection with habits, a special reference is made to the fact that the type of alimentation and the effects of masticatory function are capable of modifying craniofacial growth. It is recognized that the incorporation of modern man’s soft diet during the last few hundred years has produced less facial growth as a response to less masticatory force.

Only recently has it been possible to demonstrate this through animal experimentation. Processed and cooked food, as compared to a raw, dry diet, causes less facial growth (Lieberman et al., 2004), and the shortening of the elevating mandibular muscle fibers (He, 2004).

Although not an objective of this study, it provided the opportunity to analyze the different temporomandibular joints of 20 humans and 12 chimpanzees.

It was found that in all the chimpanzee specimens, the articular tubercle, although flatter than in humans, is concave-convex as corresponds to a temporal-disk joint that is reciprocal whilst the mandibular condyle is larger than in humans. The disk-condylar joint is of a condylar nature. This arrangement is similar to that of other omnivores. Herbivores have an inverted arrangement. The temporal-disk joint is condylar whereas the disk-condylar joint is reciprocal (Bermejo-Fenoll et al., 1993).

### Genetics

Genetics is another important aspect of the factors that influence craniofacial development and growth.

There are at least 19 genes that influence facial morphology. The genotype is regulated at many different levels, including the epigenetic level that conditions the facial phenotype. Future multidisciplinary studies are necessary for a better understanding of the genetic complexity of human facial variations (Roosenboom et al., 2016). At a chromosomic level, chromosome 18, or trisomy 21 disorders can produce facial development changes and instability (Starbuck et al., 2017). A relationship has been found between malocclusions (Angle class 1, 2, and 3) and certain genotypes (Fontoura et al., 2015) and between growth patterns and facial muscle activity (Alabdullah et al., 2015).

### Speech

Speech, although incorporated later, must have been a determining factor in the evolution of the oral bone structures during the last thousands of years.

In our society, it has been estimated that a person pronounces an average of 16,000 words a day (Mehl et al., 2007). This means the generation of over 30,000 syllables or simple sounds in a 24-h period that in turn are associated with an equivalent number of movements of the tongue, lips, and joints that connect the mandible to the skull. On the other hand, it is estimated that people perform an average of 900 chewing movements in a period of 1 h while eating (Päßler and Fischer, 2014) – representing an activity level far lower than that found in the formation of words.

The base sound or laryngeal tone originates in the vocal cords, which have morphological characteristics that are similar among different species. The formation and modulation of words basically take place in the cavities and elements of the upper vocal tract (pharynx, oral cavity, nostrils, and paranasal sinuses). In this regard, frequency, rhythm, tone, and timbre are modulated by the tongue, teeth, lips, resonating cavities, and temporomandibular joint movements.

It is not easy to find another human task so widely used throughout mankind’s existence as the “invention of syllables.”

Many studies have investigated speech in ancient humans. The main lines of research have focused on the obtainment of endocasts of Broca’s area for the purpose of analyzing the language zone in the left cerebral hemisphere (Bruner, 2017); study of the skull base, its flexion and the displacements of the foramen magnum, in an attempt to obtain information on the vocal tract or phonatory apparatus (Lieberman, 2007); investigation of the orifice in the skull base through which the hypoglossal nerve penetrates to innervate the muscles of the tongue (Jungers et al., 2003); study of the hyoid bone, lying free at the base of the tongue (Capasso et al., 2008); investigation of the middle ear, linking hearing to speech motor activity (Stoessel et al., 2016); and the analysis of the FOX P2 gene, which is related to speech, among other functions (Lieberman, 2006; Konopka et al., 2009).

## Objectives

1. To measure those anthropometric parameters that define the structures related to the oral cavity and which are linked to speech, describing both classical and new variables, in dry skulls of humans and chimpanzees.

2. To identify those variables related to speech that best differentiate between modern humans and chimpanzees, and to quantify the observed differences.

## Materials and Methods

A sample of dry adult Caucasian skulls (*n* = 20) and mandibles (*n* = 20) was obtained from the collection belonging to the Miguel Hernández University (Elche, Spain). A second sample of dry chimpanzee skulls (*n* = 12) and mandibles (*n* = 12) was obtained from the collection belonging to Valladolid University Faculty of Medicine (Spain). The gender distribution was 50% of each. The material from the collections belonging to the Faculties of Medicine of both Elche and Valladolid Universities has been obtained in accordance with international ethical standards. The regulations set by the journal have also been observed.

All the specimens were in perfect condition, without apparent deformities, fractures or asymmetries. The presence of alveoli without teeth was taken to represent postmortem tooth loss, and the odontogram only recorded the teeth that were present.

All measurements were made by the same observer using the same typical anthropometric instruments (calipers, goniometer) with sufficient precision to guarantee a direct measurement error far lower (<10%) than the standard deviations of the samples.

Nine variables for each skull and 28 variables for each mandible were defined. A total of 37 parameters was thus recorded (Table 1). The variables corresponding to the hard structures related to the temporomandibular joints were coded as numbers 10 and 13, while the variables corresponding to the hard structures related to the tongue were coded as numbers 9 and 12. In turn, variable 11.14 was related to the chin, and variables 11.4, 11.5, 11.6, 11.7, and 11.15 were related to insertion of the temporal muscle in the mandible.

**Table 1:** Mean and standard deviation (SD) of the study variables in humans and chimpanzees (Student’s *t*-test).

Anatomical structure	Description of the variable	Figures	CODE Humans			Chimpanzees			
				Mean	*SD*	Mean	*SD*	*P*-value
Palatal Space in relation to the tongue.	Length^∗∗^	1A	9.1	27.6	4.8	42.3	3.3	<0.001^∗^
	Width	1A	9.2	32.9	3.2	36.3	7.7	0.1
	Height	1A	9.3	13.4	2.4	15.5	7.9	0.3
	Total palate length	1A	9.4	45.8	3.8	77.9	7.2	<0.001^∗^
Articular Tubercle of the temporal bone in relation to the temporomandibular joint.	Length	1A	10.1	12.5	2.2	20.6	3.0	<0.001^∗^
	Width	1A	10.2	24.6	3.1	27.9	3.2	0.007^∗^
	Height	1A	10.3	8.0	2.0	2.1	1.5	<0.001^∗^
	Length/width^∗∗∗^	–	10.4	0.5	0.1	0.7	0.1	<0.001^∗^
	Height/total palate length	–	10.5	0.17	0.05	0.03	0.02	<0.001^∗^
Mandible	Length	1C	11.1	118.5	4.0	139.4	9.2	<0.001^∗^
	Bicondylar width	1B	11.2	110.0	8.2	106.8	8.4	0.3
	Bigonion width	1C	11.3	87.9	8.4	97.2	13.2	0.044^∗^
	Condyle-ramus height	1B	11.4	64.3	6.2	66.8	10.1	0.4
	Sigmoid-ramus height	1B	11.5	50.5	5.4	56.4	7.7	0.016^∗^
	Coronoid-ramus height	1B	11.6	67.9	8.2	71.2	8.8	0.3
	Sigmoid/coronoid height	–	11.7	0.8	0.1	0.8	0.1	0.1
	Ramus length	1B	11.8	30.8	2.3	47.1	4.4	<0.001^∗^
	Body thickness	1B	11.9	10.8	1.3	15.2	2.2	<0.001^∗^
	Body height	1B	11.10	28.5	4.2	34.0	2.6	<0.001^∗^
	Robustness index (thickness x 100/height)	–	11.11	40.4	9.4	45.3	9.5	0.2
	Mandibular angle^∗∗∗∗^	1B	11.12	119.6	15.9	109.2	9.4	0.048^∗^
	Divergence angle	1C	11.13	51.8	9.9	47.8	7.0	0.2
	External slope of the mandibular symphysis	1B, 3E, 3F	11.14	62.8	10.6	116.1	7.1	<0.001^∗^
	Coronoid angle	1B, 2A, 2B	11.15	47.4	7.9	61.5	11.7	<0.001^∗^
Mandibular Space in relation to the tongue.	Internal slope of the mandibular symphysis	1B, 3C, 3D	12.1	89.3	7.6	126.6	26.8	<0.001^∗^
	Length	1C	12.2	25.7	4.7	38.8	3.9	<0.001^∗^
	Width	1C	12.3	34.3	4.7	35.3	2.0	0.5
	Height	1C	12.4	12.9	3.4	15.0	3.9	0.1
	Mandibular alveolar vergency	3A, 3B	12.5	80.7	18.2	119.0	10.3	<0.001^∗^
Mandibular Condyle in relation to the temporomandibular joint.	Length	1B	13.1	8.6	1.9	13.1	4.7	0.001^∗^
	Width	1B	13.2	19.9	2.7	24.6	2.3	<0.001^∗^
	Height	1B	13.3	16.2	4.0	24.7	4.0	<0.001^∗^
	Bicondylar angle	1C	13.4	141.7	10.6	147.3	20.0	0.4
	Anterior surface AS	1B	13.5	4.4	1.1	5.5	1.4	0.035^∗^
	Posterior surface PS	1C	13.6	8.3	2.1	11.0	3.1	0.013^∗^
	AS/PS ratio	–	13.7	0.6	0.2	0.5	0.2	0.5
	Condylar angle	4A, 4B	13.8	84.9	11.3	112.3	13.1	<0.001^∗^

*^∗^p < 0.05; ^∗∗^Lengths in mm; ^∗∗∗^Adimensional ratios; ^∗∗∗∗^Angles in degrees. Underlined: greatest mean value.*

**FIGURE 1:**
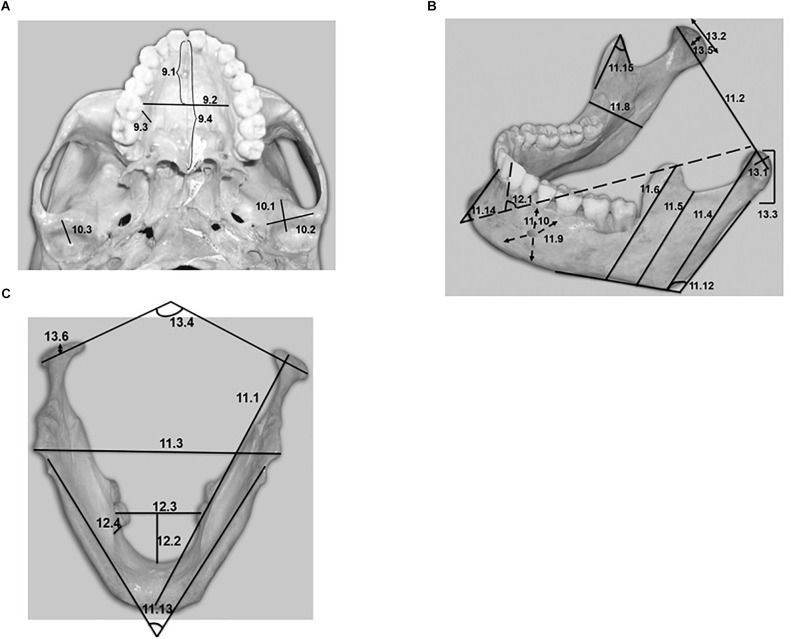
**(A)** Basal view of a human skull showing the variables encoded as 9 and 10 in Table 1. **(B,C)** Lateral and inferior view of a human mandible showing the variables encoded as 11, 12, and 13 in Table 1.

**FIGURE 2:**
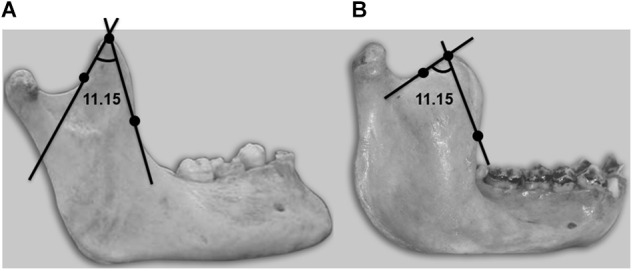
Lateral view of a mandible showing the coronoid angle, with its three defining references: vertex and anterior and posterior points, where the convexity becomes concave. **(A)** Human. **(B)** Chimpanzee.

**FIGURE 3:**
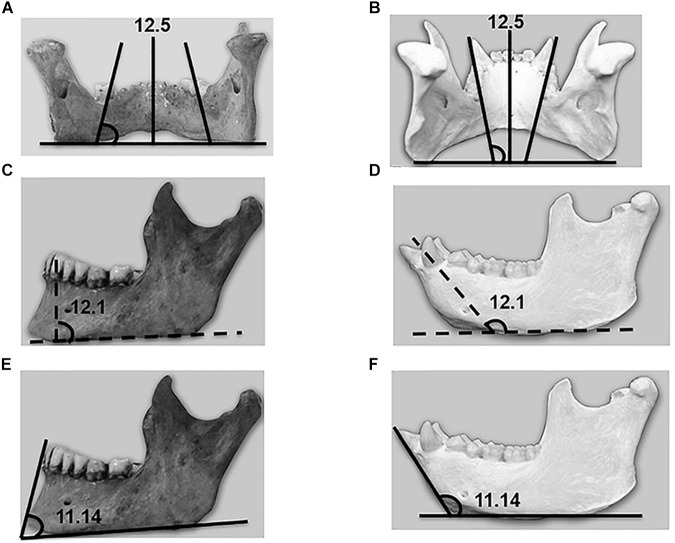
**(A)** Human. Convergence is observed toward the palate on the part of the lingual wall of the mandibular alveoli at lower first molar level, above the mylohyoid line. **(B)** Chimpanzee. Divergence is observed toward the palate on the part of the lingual wall of the mandibular alveoli at lower first molar level, above the mylohyoid line. **(C)** Human. Schematic representation of the internal slope of the mandibular symphysis. **(D)** Chimpanzee. Schematic representation of the internal slope of the mandibular symphysis. **(E)** Human. Schematic representation of the external slope of the mandibular symphysis. **(F)** Chimpanzee. Schematic representation of the external slope of the mandibular symphysis.

**FIGURE 4:**
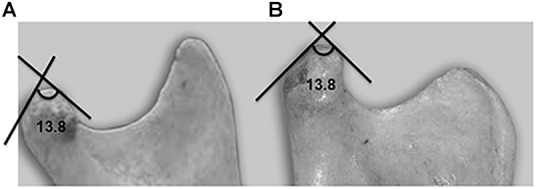
Lateral view of a mandible showing the condylar angle. **(A)** Human. **(B)** Chimpanzee.

All the measured parameters are graphically represented in Figure 1–4.

Although the teeth intervene in the pronunciation of words, no variables related to them were proposed. The frequency of postmortem tooth loss and the fact that the literature does not take the teeth into account when analyzing craniometric points supported their exclusion from the analysis (Azevedo et al., 2012).

A photographic register was obtained of all the specimens under uniform conditions using a Sony camera (8.1 megapixels) with a Carl Zeiss lens (2.8–5.4/7.9–23.7, Vario-Tessar).

Casts were obtained of each articular tubercle of the temporal bone and of the mandibular condyle, using high precision vinylpolysiloxane dental imprint material (Virtual^®^ Light Body Regular Set, Germany).

Data analysis was carried out with the SPSS version 18 statistical package.

## Results

Fifty percentage of both the 20 human and 12 chimpanzee specimens were male. All the variables exhibited a normal distribution; the Student’s *t*-test was therefore used for the comparison of mean values between species.

In the palatal space, significant differences between humans and chimpanzees were limited to length, which was seen to be greater in the latter species. In the case of the articular tubercle of the temporal bone, significant differences were recorded for all the parameters, with higher values referred to length, width and length/width ratio in chimpanzees, and comparatively greater height in humans. At mandibular level, significant differences were observed in 8 of the 14 parameters (57%), with higher values in chimpanzees in all cases except bicondylar width, which was greater in humans. In the mandibular space, significant differences were recorded in symphysis slope, length and alveolar vergency, with higher values in chimpanzees in all cases. Lastly, differences were recorded in 6 of the 8 parameters referring to the mandibular condyle, with comparatively higher values in chimpanzees in all cases.

Table 1 shows the mean values of the parameters, with the corresponding standard deviations (lengths being expressed in mm and angles in degrees). The statistical significance of the differences between the two species is also shown (statistical significance being considered for *p* < 0.05).

Of the 37 variables included in the study, 25 showed significant differences between humans and chimpanzees (with *p* < 0.001 in 18 of them). 31 parameters were of greater magnitude in chimpanzees than in humans. Only the height of the articular tubercle (variable 10.3), the bicondylar width of the mandible (variable 11.2), the mandibular angle (variable 11.12) and the mandibular divergence angle (variable 11.13), were greater in humans. Variables 11.7 and 13.7 showed very similar values in both species.

New variables were also recorded, the most important being the coronoid angle (variable 11.15), the mandibular alveolar vergency (variable 12.5) and the condylar angle (variable 13.8).

## Discussion

### Key Results

(a) The human temporomandibular joint is comparatively less flat and has a more limited excursive movement range, with lighter structural components.
(b) Significant differences were observed between the two species in mandibular alveolar vergency and in the internal slope of the mandibular symphysis that modify the oral cavity and also increase the free space for tongue movements.
(c) The appearance of the chin in humans, the only mammal that possesses one, suggests a modifying muscular stimulant of the anterior mandibular body. Using the Spanish population as reference, humans have a 56% greater body mass compared with chimpanzees: about 70 kg in humans (Encuesta Europea de Salud en España, 2009) versus 44 kg in chimpanzees (Jones et al., 1996). Therefore it is logical for the measurements reflected in Table 1 to be greater in humans. However, the opposite is generally observed. Of 24 length measurements, 22 were found to be greater in chimpanzees, and only the height of the articular tubercle and bicondylar width were greater in humans. This surprising finding suggests that the evolution of the oral zone in humans has tended toward a reduction in size, with changes in shape.

When speaking, multiple structures function in both the upper and lower vocal tracts.

These bones, ligaments, cartilages, and muscles function due to the activity of cranial nerves V, VII, IX, X, and XII.

Three main elements in the mouth are set in motion when a word is pronounced: the temporomandibular joints, the tongue and the lips.

### The Temporomandibular Joints

The morphology of the human temporomandibular joints is complex. These are not normal load-bearing joints as generally found elsewhere in the body, since their surfaces are covered with fibrous tissue, and they undergo millions of movements in the context of speech, without force transmission – acting as “floating” joint surfaces. During speech, the temporomandibular joint serves as a joining element between the mandible and skull, not as a fulcrum for applying leverage.

In all mammals except for herbivores the temporal-disk joint is a reciprocal joint and the disk-condylar joint is of a condylar nature (Bermejo-Fenoll et al., 1987, 1992; Bermejo et al., 2002).

On the other hand, in herbivores the temporal-disk joint is condylar and the disk-condylar joint is reciprocal (Bermejo-Fenoll et al., 1993).

Herbivores are characterized by very flat and extensive joint surfaces, adapted to the chewing movements inherent in a plant diet.

The comparative analysis of the temporal surface between humans and chimpanzees revealed interesting differences in length (variable 10.1) and width (variable 10.2); both were far greater in chimpanzees, while height (variable 10.3) was greater in humans (Figure 1A), with important variations. The temporal joint surface was found to be flatter and larger in chimpanzees, as reported elsewhere (Lockwood et al., 2002). This is consistent with the observations referring to the temporomandibular joints of herbivores, and is related to the diet of chimpanzees.

The described characteristics allow much wider mandibular displacements in chimpanzees than in humans. Masticatory function is therefore highly versatile in the former, and the teeth can serve as a genuine toolbox. In comparison, the condylar movements required by humans for speech are very numerous but are also limited in terms of protrusion and lateralization compared with chimpanzees. This in turn is consistent with smaller temporal-disk joint surfaces and a greater height of the articular tubercle of the temporal bone, with greater depth of the mandibular fossa. During normal mandibular movement in humans, the mandibular condyle usually does not extend beyond the articular tubercle of the temporal bone (Muto et al., 1994). Therefore, observed differences may be explained in terms of movements that are more numerous but also more limited in space in humans compared with chimpanzees.

The joint that is positioned between the articular meniscus and the jaw is a synovial joint of a condylar nature in both chimpanzees and humans. The mandibular joint surface is formed by the mandibular head or condyle, an ellipsoid with one lateral and one medial pole.

The condyle joint surface is convex on all sides and has an anterior and a posterior slope. These are separated by a transversal “crest” or ridge that crowns it lateromedially like a diadem.

Eight variables relating to the mandibular condyle were studied as referred to in Table 1, encoded 13.

In general, it can be said that the condyle in chimpanzees is much larger than in humans. The length, width and height measurements are significantly greater in all cases (*p* ≤ 0.001).

The significant variable, denominated “condylar angle” is formed by the tangents at the anterior and posterior slopes of the joint surface at the highest point of the “crest” or ridge (Figure 4). When the two species were compared, a significant difference (*p* < 0.001) was found, with the angle in humans being more acute. This result is coherent with what was found in relation to variable 10.3, i.e., the height of the articular tubercle, which is the same as the depth of the mandibular fossa, is greater in humans.

Thus, the mandibular condyle is flatter and the mandibular fossa is shallower in chimpanzees, due to excursive mandibular movements.

Another variable intimately related to joint movements is the coronoid angle (variable 11.15). The temporal muscle inserts in the coronoid process, and the mentioned variable allows us to evidence and quantify coronoid morphological differences between humans and chimpanzees that are noticeable to the naked eye. This angle is clearly defined by three recognizable anatomical points: the vertex and the point of inflection of the anterior and posterior margins. Very significant interspecies differences were recorded for this variable, with more acute angles in humans. The observed differences (with a finer coronoid structure in humans) could be attributable to participation of the temporal muscle in phonatory function.

Although all the masticatory muscles act upon the temporomandibular joints (Saifuddin et al., 2001), the temporal muscles play a special role in speech, and the coronoid process is consequently thinner in humans (Figure 2). In the chimpanzee, the coronoid process of the mandible has a comparatively rougher structure. In this species, the temporal muscles appear to participate in mandibular excursion movements and in elevator action at the moment of maximum muscle strength as in the case of the masseter and medial pterygoid muscles. The refined structure of the human coronoid process would allow optimum torque adjustment upon contraction of the temporal muscle, facilitating the fine mandibular movements inherent in speech function.

Since humans consume a softer diet, their musculoskeletal structures no longer generate forces as intense as those produced in chimpanzees, with consequent savings in terms of bone and muscle mass. This fact and the great number of movements that occur during speech involve substantial energy savings.

On the other hand, the greater length of the palate in chimpanzees (variable 9.1) is coherent with the greater length of the mandible in this species (variable 11.1). Likewise, the greater magnitudes of variables 11.7, 11.8, and 11.9 are consistent with the greater need for masticatory force transmission in chimpanzees.

### The Tongue

The oral cavity is a sensory region that houses the tongue. Forces are transmitted through the teeth, and in this way the oral cavity constitutes a free space. The tongue has a number of functions, including taste sensation, licking action, food bolus distribution between the dental arches during chewing, cleaning of the dental and mucosal surfaces with the filiform papillae, swallowing and – in humans – speech function. In comparison with chimpanzees, the human tongue has lost some functions, such as the capacity to trap and drink water by licking.

The fact that hominids at some point learned how to speak undoubtedly established differences with chimpanzees that are effectively reflected in the hard structures related to the tongue, i.e., the internal portion of the mandible.

Two parameters revealed interesting differences: variable 12.5, which has been called “mandibular alveolar vergency,” and variable 12.1, which has been called “internal slope of the mandibular symphysis.” Both parameters refer to the mandibular lingual space, with highly significant differences between humans and chimpanzees. In relation to mandibular alveolar vergency, and as can be seen in Figure 3A,B, humans are characterized by convergence toward the palate of the lingual wall of the mandibular alveoli at lower first molar level, with angles generally less than 90 degrees with respect to the vertical. In contrast, chimpanzees are characterized by divergence toward the palate, with angles generally greater than 90 degrees with respect to the vertical. The difference in this variable between the two species was found to be very significant (*p* < 0.001).

Studies related to this subject can be found in dental literature, though limited to the teeth. In this regard, on analyzing human occlusion, the lingualized position of the crowns of the lower molars causes the lingual cuspids to be located a little lower than the buccal cuspids. This results in the so-called Wilson curve (Guichet, 1977), which can be defined as the curve traced along the buccal and lingual cuspids of the upper and lower molars and premolars. In terms of dental occlusion, this curve would imply greater masticatory efficacy.

The above situation is the partial consequence of a more general process affecting the entire oral cavity. Humans require free space above the dorsal lingual surface in order to facilitate pronunciation. This free space allows the molars to incline toward the sagittal and middle planes.

Although not an objective of the study, the Wilson curve was found to be inverted in the 12 chimpanzee mandibles with respect to the situation found in humans, i.e., its concavity is oriented as corresponds to the distribution of the molars according to the afore mentioned mandibular alveolar vergency.

The importance of the above-mentioned free space for the pronunciation of words becomes manifest when the space is lost, such as, for example, on fitting palatal dental prostheses or palatal orthodontic appliances, in cases of macroglossia, or in individuals with Down syndrome (Panchón-Ruiz et al., 2000; Raposo et al., 2011). In all three scenarios pronunciation is altered, particularly lip-palatal and lip-dental syllables.

The evolution of the human palate toward a more spherical shape compared with the chimpanzee can be interpreted in a similar way. By shortening its length (variable 9.1) 21% and keeping its width (variable 9.2), and height (variable 9.3) approximately the same, the palate experiences relative widening, changing from a paraboloid shape to a more elliptic shape.

Variable 12.1 (“internal slope of the mandibular symphysis”) is intimately related to this evolutive process. The differences between humans and chimpanzees were very significant (*p* < 0.001). Figure 3C,D show the difference in the angle between the two species, ranging from 126.6 degrees in chimpanzees to 89.3 degrees in humans. Verticalization of the mandible and teeth was observed that proved consistent with the evolutive trend reflected by the previously discussed mandibular alveolar vergency.

### The Lips

Lastly, major differences were observed between the two species in the variable referred to as the “external slope of the mandibular symphysis” (variable 11.14), which is defined as the angle formed between the line joining the infradentale point with the most prominent portion of the chin and the horizontal line crossing the lower part of the mandible. The differences were very significant not only quantitatively (*p* < 0.001) but also qualitatively (Figure 3E,F).

Chin prominence evidences this variable which is so representative of humans.

Scientific literature offers a number of theories to account for the existence of the human chin. Some authors regard it as a mandibular reinforcing structure designed to facilitate the crushing of food (Gröning et al., 2011); or a facial restructure due to new eating habits where the facial middle third is reduced and the lower third relatively more advanced (Holton and Franciscus, 2008). In turn, it has been postulated that reduction of the facial middle third and the persistence of tongue size would force reorganization of the vocal tract to allow breathing and swallowing – thereby leading to formation of the chin or mentalis prominence (Coquerelle et al., 2013). Lastly, some authors consider that the chin affords protection against the stress caused by micro movements of the tongue (Ichim et al., 2007).

In the present study, the characteristic human chin has been linked to the appearance of speech, and is postulated to have developed as a result of the persistent action of the mentalis muscle upon the mandibular symphysis in the context of the phonatory process.

The mentalis muscle is a bilateral structure. Each muscle (right and left) extends from the chin (on either side of the mandibular symphysis) anteriorly and penetrates the region of the orbicular, quadrate, and triangular muscles. It does not specifically extend to the lower lip but rather to the muscles that mobilize the lip. In this regard it acts as an antigravity muscle. The mentalis muscle depresses the lower lip muscles against the inferior alveoli, allowing them to function properly and contributes to upper and lower lip coaptation during chewing and speech.

No analogous muscle is found in the upper lip, since gravity performs its function. Coaptation of the lips is crucial in all mammals as it prevents food from falling out of the mouth during chewing. While chimpanzees also have this mentalis muscle (Diogo et al., 2013), it is comparatively hypertrophic in humans, as evidenced by the team’s previous dissections.

It is logical to assume that this morphology has its origin in speech function. Coaptation of the lips is necessary for pronouncing bilabial syllables. As a result, in humans the mentalis muscle is continuously very active in its dual eating and speech facilitating function. Such activity can be expected to stimulate bone formation at the point of insertion of the muscle and this may have given rise to the chin (conforming a genuine chin process) – making *H. sapiens* the only mammalian species with this anatomical feature. Humans are also unique in having a red labial margin, which may have appeared when the species started to pronounce bilabial syllables.

New theories have recently been proposed in an attempt to explain the great technological and cultural leap that took place about 50,000 years ago (Cieri et al., 2014). There is now awareness of the influence of speech and written language upon brain development (Huth et al., 2016; Lallier and Carreiras, 2018). In this regard, there are important differences in brain structure between modern humans and chimpanzees, characterized by differentiation from a common ancestor (Gómez-Robles et al., 2014).

On the other hand, it is known that the lower vocal tract (comprising the vocal cords and the rest of laryngeal elements) is very similar in apes and humans (Fitch et al., 2016; Lameira et al., 2016), with both species having similar mechanisms for orofacial movements (Toyoda et al., 2017). However, on the basis of the results obtained, it can be concluded that there is a difference between the oral cavity of humans and that of chimpanzees.

Throughout this discussion the team has tried to find reasonable explanations for the results obtained.

The bone structures of the human mouth probably co-evolved with the spoken language, although the team is also aware of the difficulty that exists in demonstrating the working hypothesis.

As the lower vocal tract is similar in both humans and chimpanzees, as has been indicated, the differences between the two species have to be sought in the upper vocal tract, as according to Rauschecker (2018) in a recent paper.

The human temporomandibular joint is comparatively less flat and has a more limited excursive movement range. There is a greater free mandible space that allows the tongue more freedom of movement. Also, there is a chin that contains the mentalis muscle which is essential for pronouncing bilabial syllables.

These are new pronouncements that had not been studied sufficiently until now.

Follow-up research and comparative studies that relate the hard structures with the soft tissue structures of the upper vocal tract are necessary to confirm the results of this study.

## Author Contributions

All authors listed have made a substantial, direct and intellectual contribution to the work, and approved it for publication.

## Conflict of Interest Statement

The authors declare that the research was conducted in the absence of any commercial or financial relationships that could be construed as a potential conflict of interest.

## References

[B1] Alabdullah M. Saltaji H. Abou-Hamed H. Youssef M. (2015). Association between facial growth pattern and facial muscle activity: a prospective-sectional study. *Int. Orthod.* 13 181–194. 10.1016/j.ortho.2015.03.011 25986702

[B2] Azevedo S. Pucciarelli H. Lanata J. L. González-José R. (2012). Identificando señales de evolución no estocástica en la morfología craneofacial de poblaciones humanas modernas. *Rev. Arg. Antropol. Biol.* 14 113–129.

[B3] Bermejo A. Panchón A. González J. González O. (2002). A study of the movements of the human temporomandibular joint complex in the cadaver. *J. Cranio. Mandib. Pract.* 20 181–191. 10.1080/08869634.2002.11746209 12150264

[B4] Bermejo-Fenoll A. González-Sequeros O. González-González J. M. (1992). Histological study of the temporomandibular joint capsule: theory of the articular complex. *Acta Anat.* 145 24–28. 10.1159/000147337 1414209

[B5] Bermejo-Fenoll A. González-Sequeros O. González-González J. M. (1993). The pig: an animal model for experimentation of the temporomandibular articular complex. *Oral Surg. Oral Med. Oral Pathol.* 75 18–23. 10.1016/0030-4220(93)90399-o 8419867

[B6] Bermejo-Fenoll A. Puchades-Orts A. Sánchez-del-Campo F. Panchón-Ruíz A. Herrera-Lara M. (1987). Morphology of the meniscotemporal part of the temporomandibular joint and its biomechanical implications. *Acta Anat.* 129 220–226. 10.1159/000146404 3661114

[B7] Bruner E. (2017). Language, paleoneurology, and the fronto-parietal system. *Front. Hum. Neurosci.* 11:349. 10.3389/fnhum.2017.00349 28713257 PMC5491953

[B8] Capasso L. Michetti E. D’Anastasio R. (2008). A homo erectus hyoid bone: possible implications for the origin of the human capability for speech. *Coll. Antropol.* 32 1007–1011. 19149203

[B9] Cieri R. L. Churchill S. E. Franciscus R. Tan J. Hare B. (2014). Craniofacial feminization, social tolerance, and the origins of behavioral modernity. *Curr. Anthropol.* 55 419–443. 10.1086/677209

[B10] Coquerelle M. Prados-Frutos J. C. Rojo R. Mitteroecker P. Bastir M. (2013). Short faces, big tongues: developmental origin of the human chin. *PLoS One* 8:e81287. 10.1371/journal.pone.0081287 24260566 PMC3829973

[B11] Diogo R. Potau J. M. Pastor J. F. de Paz F. J. Ferrero E. M. Bello G. (2013). *Photographic and Descriptive Musculoskeletal Atlas of Chimpanzees.* London, FL: CRC Press.

[B12] Encuesta Europea de Salud en España (2009). *INE (Instituto Nacional de Estadística).* Available at: www.ine.es

[B13] Fitch W. de Boer B. Mathur N. Ghazanfar A. (2016). Monkey vocal tracts are speech-ready. *Sci. Adv.* 2:e1600723. 10.1126/sciadv.1600723 27957536 PMC5148209

[B14] Fontoura C. S. Miller S. F. Wehby G. L. Amendt B. A. Holton N. E. Southard T. E. (2015). Candidate gene analyses of skeletal variation in malocclusion. *J. Dent. Res.* 94 913–920. 10.1177/0022034515581643 25910506 PMC4530344

[B15] Gómez-Robles A. Hopkins W. Sherwood C. (2014). Modular structure facilitates mosaic evolution of the brain in chimpanzees and humans. *Nat. Commun.* 5:4469. 10.1038/ncomms5469 25047085 PMC4144426

[B16] Gröning F. Liu J. Fagan M. J. O’Higgins P. (2011). Why do humans have chins? Testing the mechanical significance of modern human symphyseal morphology with finite element análisis. *Am. J. Phys. Anthropol.* 144 593–606. 10.1002/ajpa.21447 21404235

[B17] Guichet N. F. (1977). *Occlusion. A Teaching Manual.* Anaheim, CA: Denar Corporation.

[B18] He T. (2004). Craniofacial morphology and growth in the ferret: effects from alteration of masticatory function. *Swed. Dent. J.* 165 1–72. 15224640

[B19] Holton N. E. Franciscus R. G. (2008). The paradox of a wide nasal aperture in cold-adapted neandertals: a causal assessment. *J. Human Evol.* 55 942–951. 10.1016/j.jhevol.2008.07.001 18842288

[B20] Huth A. de Heer W. Griffiths T. Theunissen F. Gallant J. (2016). Natural speech reveals the semantic maps that tile human cerebral cortex. *Nature* 532 453–458. 10.1038/nature17637 27121839 PMC4852309

[B21] Ichim I. Kieser J. Swain M. (2007). Tongue contractions during speech may have led to the develpment of the bony geometry of the chin following the evolution of human lenguaje: a mechanobiological hypothesis for the development of the human chin. *Med. Hypotheses* 69 20–24. 10.1016/j.mehy.2006.11.048 17280797

[B22] Jones C. Jones C. Jones J. Wilson D. (1996). Pan troglodytes. *Mammalian Species* 529 1–9. 10.1016/j.ijpp.2012.06.002 29539346

[B23] Jungers W. L. Pokempner A. A. Kay R. F. Cartmill M. (2003). Hypoglossal canal size in living hominoids and the evolution of human speech. *Hum. Biol.* 75 473–484. 10.1353/hub.2003.0057 14655872

[B24] Konopka G. Bomar J. M. Winden K. Coppola G. Jonsson Z. Gao F. (2009). Human-Specific transcriptional regulations of CNS development genes by FOXP2. *Nature* 462 213–217. 10.1038/nature08549 19907493 PMC2778075

[B25] Lallier M. Carreiras M. (2018). Cross-linguistic transfer in bilinguals reading in two alphabetic orthographies: the grain size accommodation hypothesis. *Psychon. Bull. Rev.* 25 386–401. 10.3758/s13423-017-1273-0 28405906

[B26] Lameira A. R. Hardus M. E. Mielke A. Wich S. Shumaker R. W. (2016). Vocal fold control beyond the species-specific repertoire in an orang-utan. *Sci. Rep.* 6:30315. 10.1038/srep30315 27461756 PMC4962094

[B27] Lieberman D. E. Krovitz G. E. Yates F. W. Devlin M. St Claire M. (2004). Effects of food processing on masticatory strain and craniofacial growth in a retrognathic face. *J. Hum. Evol.* 46 655–677. 10.1016/j.jhevol.2004.03.005 15183669

[B28] Lieberman D. E. McCarthy R. C. Hiiemae K. M. Palmer J. B. (2001). Ontogeny of postnatal hyoid and larynx descent in humans. *Arch. Oral Biol.* 46 117–128. 10.1016/s0003-9969(00)00108-4 11163319

[B29] Lieberman P. (2006). The FOXP2 gene, human cognition and language. *Int. Congr. Ser.* 1296 115–126. 10.1016/j.ics.2006.03.039

[B30] Lieberman P. (2007). The evolution of human speech: its anatomical and neural bases. *Curr. Anthropol.* 48 39–66. 10.1086/509092 22106428

[B31] Lockwood C. A. Lynch J. M. Kimbel W. H. (2002). Quantifying temporal bone morphology of great apes and humans: an approach using geometric morphometrics. *J. Anat.* 201 447–464. 10.1046/j.1469-7580.2002.00122.x 12489757 PMC1570994

[B32] Mehl M. R. Vazire S. Ramírez-Esparza N. Slatcher R. B. Pennebaker J. W. (2007). Are women really more talkative than men? *Science* 6:82. 10.1126/science.1139940 17615349

[B33] Muto T. Kohara M. Kanazawa M. Kawakami J. (1994). The position of the mandibular condyle at maximal mouth opening in normal subjects. *J. Oral Maxillofac. Surg.* 52 1269–1272. 10.1016/0278-2391(94)90049-3 7965330

[B34] Nishimura T. Mikami A. Suzuki J. Matsuzawa T. (2006). Descent of the hyoid in chimpanzees: evolution of face flattening and speech. *J. Human Evol.* 51 244–254. 10.1016/j.jhevol.2006.03.005 16730049

[B35] Panchón-Ruiz A. Jornet-Carrillo V. Sanchez del Campo F. (2000). Palate vault morphology in down syndrome. *J. Craniofac. Genet. Dev. Biol.* 20 198–200. 11354516

[B36] Päßler S. Fischer W. J. (2014). Food intake monitoring: automated chew event detection in chewing sounds. *IEEE J. Biomed. Health Inform.* 18 278–289. 10.1109/JBHI.2013.2268663 24403426

[B37] Raposo A. Preisler G. Salinas F. Muñoz C. (2011). Glossoplasty with harada’s technique in a patient with down syndrome. *Int. J. Odontostomat.* 5 245–248.

[B38] Rauschecker J. P. (2018). Where did language come from? Precursor mechanisms in nonhuman primates. *Curr. Opin. Behav. Sci.* 21 195–204. 10.1016/j.cobeha.2018.06.003 30778394 PMC6377164

[B39] Roosenboom J. Hens G. Mattern B. Shriver M. Claes P. (2016). Exploring the underlying genetics of craniofacial morphology through various sources of knowledge. *Bio. Med. Res. Int.* 2006:3054578. 10.1155/2016/3054578 28053980 PMC5178329

[B40] Saifuddin M. Miyamoto K. Ueda H. M. Shikata N. Tanne K. (2001). A quantitative electromyographic analysis of masticatory muscle activity in usual daily life. *Oral Dis.* 7 94–100. 10.1034/j.1601-0825.2001.0070205.x 11355445

[B41] Shanhraki N. Yassaei S. Goldani M. (2012). Abnormal oral habits: a review. *J. Dent. Oral Hyg.* 4 12–15.

[B42] Starbuck J. M. Cole T. M. Reeves R. H. Richsmejer J. T. (2017). The influence of trisomy 21 on facial form and variability. *Am. J. Genet. A* 173 2861–2872. 10.1002/ajmg.a.38464 28941128 PMC5679727

[B43] Stoessel A. Gunz P. David R. Spoor F. (2016). Comparative anatomy of the middle ear ossicles of extant hominids - Introducing a geometric morphometric protocol. *J. Hum. Evol.* 91 1–25. 10.1016/j.jhevol.2015.10.013 26852810

[B44] Toyoda A. Maruhashi T. Malaivijitnond S. Koda H. (2017). Speech-like orofacial oscillations in stump-tailed macaque (Macaca arctoides) facial and vocal signals. *Am. J. Phys. Anthropol.* 164 435–439. 10.1002/ajpa.23276 28681947

